# Evolutionary Trajectory of *Plasmodium falciparum*: From Autonomous Phototroph to Dedicated Parasite

**DOI:** 10.3390/biomedicines13092287

**Published:** 2025-09-17

**Authors:** Damian Pikor, Mikołaj Hurla, Alicja Drelichowska, Małgorzata Paul

**Affiliations:** 1 Department of Internal Medicine and Metabolic Disorders, University of Medical Sciences, Przybyszewskiego 49, 60-355 Poznan, Poland; 2 Department of Tropical and Parasitic Diseases, Central University Hospital, Przybyszewskiego 49, 61-701 Poznan, Poland; 3 The Student Scientific Society of Poznan University of Medical Sciences, Rokietnicka 5, 60-806 Poznan, Poland

**Keywords:** malaria, host–pathogen co-evolution, insecticide resistance

## Abstract

Malaria persists as a paradigmatic model of co-evolutionary complexity, emerging from the dynamic interplay among a human host, Anopheles vectors, and *Plasmodium falciparum* parasites. In human populations, centuries of selective pressures have sculpted an intricate and heterogeneous immunogenetic landscape. Classical adaptations, such as hemoglobinopathies, are complemented by a diverse array of genetic polymorphisms that modulate innate and adaptive immune responses. These genetic traits, along with the acquisition of functional immunity following repeated exposures, mitigate disease severity but are continually challenged by the parasite’s highly evolved mechanisms of antigenic variation and immunomodulation. Such host adaptations underscore an evolutionary arms race that perpetually shapes the clinical and epidemiological outcomes. Intermediaries in malaria transmission have evolved robust responses to both natural and anthropogenic pressures. Their vector competence is governed by complex polygenic traits that affect physiological barriers and immune responses during parasite development. Recent studies reveal that these mosquitoes exhibit rapid behavioral and biochemical adaptations, including shifts in host-seeking behavior and the evolution of insecticide resistance. Mechanisms such as enhanced metabolic detoxification and target site insensitivity have emerged in response to the widespread use of insecticides, thereby eroding the efficacy of conventional interventions like insecticide-treated bed nets and indoor residual spraying. These adaptations not only sustain transmission dynamics in intervention saturated landscapes but also challenge current vector control paradigms, necessitating the development of innovative, integrated management strategies. At the molecular level, *P. falciparum* exemplifies evolutionary ingenuity through extensive genomic streamlining and metabolic reconfiguration. Its compact genome, a result of strategic gene loss and pruning, is optimized for an obligate parasitic lifestyle. The repurposing of the apicoplast for critical anabolic functions including fatty acid, isoprenoid, and haem biosynthesis highlights the parasite’s ability to exploit host derived nutrients efficiently. Moreover, the rapid accumulation of mutations, coupled with an elaborate repertoire for antigenic switching and epigenetic regulation, not only facilitates immune escape but also accelerates the emergence of antimalarial drug resistance. Advanced high throughput sequencing and functional genomics have begun to elucidate the metabolic epigenetic nexus that governs virulence gene expression and antigenic diversity in *P. falciparum*. By integrating insights from molecular biology, genomics, and evolutionary ecology, this study delineates the multifaceted co-adaptive dynamics that render malaria a recalcitrant global health threat. Our findings provide critical insights into the molecular arms race at the heart of host–pathogen vector interactions and underscore promising avenues for the development of next generation therapeutic and vector management strategies aimed at sustainable malaria elimination.

## 1. Introduction

**Figure 1:**
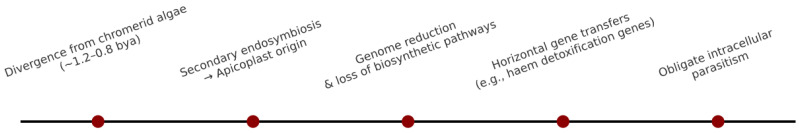
Evolutionary Timeline of *Plasmodium falciparum*.

Malaria endures as one of the preeminent infectious diseases, exacting an immense toll in morbidity and mortality across the globe [1,2]. At the heart of this enduring public health challenge is the genus *Plasmodium* with unicellular eukaryotic parasites that have evolved sophisticated strategies to infect both human and animal hosts. Recent advances in high-throughput sequencing and single-cell genomic methodologies have profoundly refined our understanding of the evolutionary history and the complex biological adaptations that underpin the success of *Plasmodium* spp. [3]. In the present essay, we integrate the evolutionary progression of *Plasmodium falciparum* into a cohesive narrative, structured around five principal thematic areas. We begin by examining the ancestral lineage and the phylogenetic positioning of *P. falciparum* within the broader alveolate assemblage, before exploring the genomic and metabolic reconfigurations that accompanied the transition to parasitism. This discussion is followed by an in-depth analysis of the molecular and structural mechanisms that govern host cell invasion and immune modulation, with a specific focus on *P. falciparum*, the species responsible for the most severe form of human malaria. Subsequently, we consider the co-adaptive dynamics between *P. falciparum* and its human hosts and mosquito vectors, with particular emphasis on the interplay of historical influences and recent genetic adaptations. Finally, the essay speculates on future research avenues, including the development of novel therapeutic strategies and the implementation of systems biology approaches in the ongoing battle against malaria. Collectively, these discussions offer a comprehensive insight into the evolutionary biology of malaria parasites and underscore their enduring significance to global health. The origins and early innovations of *P. falciparum* are illuminated by recent comparative genomic studies, which suggest that apicomplexans, including *P. falciparum*, share a profound evolutionary heritage with chromerid algae organisms that, despite their modern divergence, still retain functional photosynthetic apparatus and flagellar structures [4,5]. Molecular clock estimates, meticulously calibrated against the fossil record, propose that the divergence between these groups occurred during the mid-Proterozoic era (circa 1.2–0.8 billion years ago) (Figure 1) [6,7]. This early evolutionary split set the stage for a cascade of adaptations that would eventually culminate in a fully parasitic lifestyle. Among the most compelling vestiges of this ancestral phototrophic past is the apicoplast, a non-photosynthetic plastid that owes its origin to a secondary endosymbiotic event involving a red algal cell. Over evolutionary time, this organelle has been repurposed to fulfill essential metabolic roles, including the biosynthesis of fatty acids, isoprenoids, and haem [8,9]. *P. falciparum*, in particular, relies heavily on the apicoplast for these crucial biosynthetic pathways, making the organelle an attractive target for antimalarial drugs. Phylogenomic analyses lend robust support to the hypothesis that the common ancestor of apicomplexans was originally a free-living, photosynthetically active organism that gradually embraced an intracellular mode of existence [10,11]. The adaptive landscape of *P. falciparum* has also been significantly shaped by the phenomenon of horizontal gene transfer (HGT). Multiple discrete events of HGT from bacterial, algal, and even fungal sources have contributed to the mosaic nature of the *Plasmodium* genome [12,13]. For instance, genes implicated in the detoxification of haem, such as those coding for histidine-rich protein (HRP-II), are thought to derive from bacterial origins, thus enabling the neutralization of toxic haem liberated during the catabolism of hemoglobin [14]. This is particularly relevant for *P. falciparum*, which rapidly digests host hemoglobin to acquire amino acids, generating a significant amount of toxic free haem. The HRP-II protein, a product of this HGT event, plays a key role in sequestering this haem, a process vital for parasite survival. In addition, the metabolic pathways operating within the apicoplast, particularly those involved in fatty acid synthesis, may represent vestiges of ancient algal gene acquisitions [5]. These exogenous genetic contributions have not only broadened the metabolic capabilities of the parasite but also exemplify the dynamic and fluid nature of gene flow during the early stages of eukaryotic evolution. Integrating molecular clock analyses with paleontological data reveals that the split between apicomplexans and their photosynthetic kin transpired roughly 800–1000 million years ago [6,10]. Within the apicomplexan lineage, several pivotal evolutionary milestones are evident, chief among them being the adoption of an intracellular lifestyle. This transition was concomitant with a marked genome reduction and the systematic abandonment of several autonomous biosynthetic pathways [12]. Detailed studies of genomic divergence illustrate that this transitional phase, characterized by an escalating reliance on host-derived nutrients, was instrumental in defining the parasitic modus operandi that typifies modern *Plasmodium species*. In summation, the evolutionary narrative of *Plasmodium falciparum* is one of profound transformation from an ancestral free-living, phototrophic entity to a highly specialized, obligate intracellular parasite. This metamorphosis has been underpinned by a series of evolutionary innovations, including the retention and repurposing of the apicoplast, recurrent episodes of horizontal gene transfer, and significant genomic streamlining. The integration of advanced molecular techniques and rigorous phylogenomic analyses continues to shed light on these processes, offering ever-deeper insights into the adaptive mechanisms that have enabled *P. falciparum* to emerge as one of the most formidable pathogenic agents affecting global public health.

## 2. Genomic and Metabolic Adaptations

The evolutionary trajectory of *Plasmodium sp*. towards an obligate parasitic lifestyle is emblematic of profound genomic and metabolic reconfigurations that have been meticulously refined to optimize survival within the host milieu. A critical hallmark of this adaptation is the pronounced genomic streamlining observed in *Plasmodium species*; while free-living relatives such as *Chromera velia* boast genomes exceeding 200 megabases, *Plasmodium* genomes are markedly compact, typically ranging between 23 and 30 megabases [15]. This substantial contraction is not indicative of genetic deterioration but rather reflects a strategic evolutionary pruning process, whereby nonessential genes have been selectively discarded in favor of retaining those that facilitate host exploitation. *P. falciparum*, in particular, has a relatively compact genome of approximately 23 megabases, underscoring its extreme specialization. For example, the loss of de novo purine synthesis pathways compels the parasite to scavenge these indispensable compounds from the host, thereby minimizing metabolic overhead and streamlining energy utilization [16,17]. Concomitant with genome reduction is a dramatic reorganization of metabolic pathways, underscoring the parasite’s reliance on host-derived nutrients. *P. falciparum* has evolved a plethora of high-affinity transport mechanisms to secure glucose as a vital energy substrate during the erythrocytic cycle, as well as sophisticated strategies to appropriate host lipids for membrane biogenesis and energy storage [18,19]. The apicoplast, a vestigial plastid of algal origin, exemplifies this metabolic repurposing; it has transitioned from an ancestral photosynthetic role to underpin critical anabolic processes, including fatty acid, isoprenoid, and haem biosynthesis (Table 1) [20]. This organellar adaptation not only illustrates the parasite’s metabolic flexibility but also underscores the evolutionary integration of exogenous nutrient exploitation with intrinsic biosynthetic capacity. Recent studies have further illuminated the nexus between metabolic sensing and epigenetic regulation in *P. falciparum*, particularly through the lens of one-carbon metabolism. The generation of S-adenosylmethionine (SAM), a pivotal methyl donor in histone methylation reactions, is intimately linked to the availability of host nutrients such as choline and methionine [21,22]. Such metabolic fluxes directly impact the epigenetic landscape, modulating histone methylation patterns that govern the expression of virulence factors, including the *var* gene family responsible for antigenic variation [23,24,25]. This is of paramount importance for *P. falciparum*, as antigenic variation through the expression of different PfEMP1 proteins encoded by the *var* genes is its primary mechanism for evading the host immune system. Elevated SAM levels have been shown to reinforce heterochromatin stability, thereby silencing subsets of *var* genes while permitting the controlled activation of others in response to environmental cues [26]. This metabolic-epigenetic interplay is further corroborated by studies demonstrating that perturbations in SAM availability, whether through genetic manipulation of SAM synthetase or alterations in the extracellular nutrient milieu, lead to marked shifts in chromatin configuration and antigenic switching dynamics [27]. Moreover, the parasite’s genomic adaptations extend to the organization of its nuclear architecture. High-resolution chromatin conformation capture techniques have revealed that silent *var* genes are sequestered within heterochromatin-rich regions proximal to the nuclear periphery, whereas the active *var* gene is localized within euchromatic domains that favor transcriptional competence [21]. This spatial segregation is mediated by key epigenetic regulators, including heterochromatin protein 1 (HP1) and specific histone modifications such as H3K9me3, which collectively orchestrate the precise temporal and spatial expression of virulence determinants [25,26]. The dynamic repositioning of chromatin within the nucleus not only facilitates rapid antigenic variation, a key survival strategy for *P. falciparum*, but also underscores the integral role of nuclear organization in synchronizing metabolic and gene regulatory processes during the parasite’s intraerythrocytic developmental cycle. In summation, the genomic and metabolic adaptations of *Plasmodium*, particularly *P. falciparum*, epitomize a highly refined evolutionary strategy, whereby a streamlined genome is complemented by an intricate network of metabolic pathways and epigenetic mechanisms that together facilitate optimal host exploitation. This multifaceted adaptation enables the parasite to precisely sense and respond to fluctuations in host nutrient availability, thereby modulating its epigenetic landscape and ensuring the rapid and reversible expression of virulence genes necessary for immune evasion. Such an integrated framework not only highlights the sophistication of *Plasmodium* biology but also delineates promising avenues for therapeutic intervention aimed at disrupting the critical interplay between metabolic sensing and epigenetic regulation, a strategy that could be particularly effective against the highly virulent *P. falciparum*.

## 3. Molecular Mechanisms of Host Interaction

The intricate dance between P. falciparum and its human host hinges on the epigenetic control of var genes, which encode the crucial *Plasmodium falciparum* erythrocyte membrane protein 1 (PfEMP1) responsible for mediating adhesion and immune evasion. This regulation is achieved through a delicate balance between activating and repressive histone modifications, ensuring that only one *var* gene is expressed at a time while others remain silent. Recent studies have revealed that the parasite’s metabolic state, particularly its one-carbon metabolism and the resulting production of S-adenosylmethionine (SAM), plays a pivotal role in modulating these epigenetic marks and driving the switch in *var* gene expression, notably impacting the *var2csa* gene. By linking metabolic cues to epigenetic regulation, these findings deepen our understanding of how *P. falciparum* adapts to the host environment.

### 3.1. Epigenetic Regulation of Var Gene Expression

Molecular interactions between *P. falciparum* parasites and their human hosts have long been recognized as pivotal in the pathogenesis of malaria. The virulence of *P. falciparum* is in no small measure attributable to its remarkable capacity to express cytoadherence proteins on the surface of infected red blood cells (iRBCs). Chief among these proteins is PfEMP1, which plays an indispensable role in mediating adhesion to vascular endothelial receptors, thereby ensuring the sequestration of parasites within the microvasculature and facilitating evasion of splenic clearance [28]. A critical aspect of host interaction also involves the initial invasion of erythrocytes, a process recently shown to be dependent on specific parasite protein–host glycan interactions, such as the binding of the cysteinerich protective antigen (CyRPA) to host receptors, which highlights an additional layer of co-evolutionary adaptation [29]. The expression of PfEMP1 is underpinned by the *var* gene family, a large multicopy ensemble comprising approximately sixty genes per parasite genome, predominantly located in subtelomeric regions [30]. This phenomenon of mutually exclusive *var* gene expression, a central mechanism for antigenic variation, is critical not only for immune evasion but also for the establishment of persistent infections [31]. Modern RNA-sequencing approaches, coupled with advanced computational pipelines, have significantly enhanced the profiling of *var* gene expression, revealing complex and structured switching patterns that were previously underestimated [32]. It is now well established that epigenetic processes govern the regulation of *var* gene expression. The unique pattern of mutually exclusive expression is sustained through a delicate interplay between transcriptionally active euchromatin and repressive heterochromatin. The single *var* gene that is actively transcribed is associated with a euchromatic configuration, whereas its silent counterparts are sequestered within condensed heterochromatin. Distinct histone modifications demarcate these chromatin states; for example, acetylation of histone H3 lysine 9 is enriched at the promoter of the active *var* gene, in contrast to the tri-methylated form that predominates at silent loci [33]. Furthermore, modifications such as H3K36me3 (histone H3 lysine 36 trimethylation) are found across both active and silent *var* regions, with additional di- and tri-methylation of H3K4 observed at active promoters [33,34]. These modifications are introduced by SET-domain-containing histone methyltransferases including PfSET1 (*Plasmodium falciparum* SET-domain histone methyltransferase (1), PfSET2 (*Plasmodium falciparum* SET-domain histone methyltransferase (2), PfSET3 (*Plasmodium falciparum* SET-domain histone methyltransferase (3), and PfSET10 (*Plasmodium falciparum* SET-domain histone methyltransferase 10) whose activity is fundamental in modulating the epigenetic landscape that underlies antigenic switching, a process essential for the parasite’s persistence despite host immune defenses [34,35,36]. Notably, recent findings have revised the role of PfSET10, identifying it primarily as an H3K18 methyltransferase, which adds another layer of regulatory specificity [37]. Of particular note is *var2csa*, which encodes a specialized PfEMP1 variant with an exclusive affinity for the placental receptor chondroitin sulfate A. Its upstream regulatory region, designated as UpsE, together with an upstream open-reading frame (uORF), imposes an additional tier of regulation by mediating translational repression even when transcription is active [38,39]. This dual level of control intimates that *var2csa* may have functions that transcend those of a mere adhesion molecule, potentially occupying a central role within the regulatory network governing *var* gene switching [40,41]. The rapid activation of *var2csa* in response to destabilization of epigenetic repression thus serves as a sensitive indicator of altered *var* gene expression dynamics (Table 2).

### 3.2. Metabolic Modulation and Experimental Evidence in Var Switching

**Figure 2:**
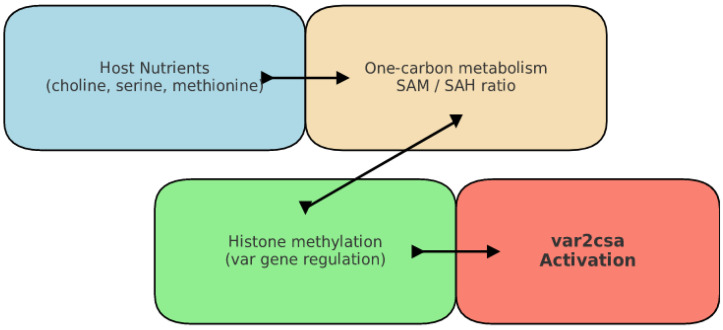
Metabolic–Epigenetic Axis Regulating Antigenic Variation.

Recent advances have revealed that the metabolic state of *P. falciparum* is inextricably linked to its epigenetic regulation. Central to this connection is the methionine arm of one-carbon metabolism, which is responsible for generating S-adenosylmethionine (SAM), the universal methyl donor essential for histone methylation reactions (Figure 2) [42]. SAM is synthesized via the folate and methionine cycles, and its subsequent conversion to S-adenosylhomocysteine (SAH), a potent inhibitor of methyltransferases, creates a finely tuned balance that is critical for appropriate methylation activity. Considering that the Michaelis constants of histone methyltransferases approximate the intracellular concentration of SAM, even modest fluctuations in the SAM/SAH ratio can have pronounced effects on the methylation of histone tails and, by extension, on gene expression [43,44,45]. Empirical evidence from diverse eukaryotic systems has further corroborated the principle that metabolic perturbations can directly remodel the epigenetic landscape [46,47,48,49,50,51]. Within the context of *P. falciparum*, recent studies have demonstrated that the parasite can modulate its gene expression in response to environmental fluctuations that alter nutrient availability, thereby influencing the intracellular SAM/SAH ratio and, consequently, *var* gene expression [52,53,54,55,56,57]. In particular, variations in the availability of lipid precursors such as choline and serine have been shown to reprogramme the flux through competing phospholipid synthesis pathways, thus impacting SAM consumption. The synthesis of phosphatidylcholine (PtdCho), the principal phospholipid of the parasite’s membrane, may proceed via the cytidine diphosphate (CDP)–choline pathway (also known as the Kennedy pathway) or via the serine decarboxylase phosphoethanolamine methyltransferase (SDPM) pathway [58,59,60]. Notably, the SDPM pathway consumes SAM during the conversion of phosphoethanolamine (p-Etn) to phosphocholine (p-Cho) by the enzyme PfPMT. Experimental manipulations that adjust the levels of choline, serine and methionine in the parasite’s milieu have yielded quantifiable changes in intracellular SAM concentrations and corresponding shifts in *var* gene expression. For instance, when parasites are cultured in media enriched with choline but depleted of serine, the resultant metabolic reprogramming favors the CDP–choline pathway over the SDPM pathway. This shift, by reducing the consumption of SAM, engenders a modest yet reproducible increase in intracellular SAM that is sufficient to disrupt the epigenetic silencing of *var* genes and prompt a coordinated switch to *var2csa* expression. Such findings have been corroborated by quantitative reverse-transcriptase PCR analyses conducted over periods of two to four weeks [36,40]. Likewise, the provision of excess methionine, which directly feeds into SAM synthesis, has been shown to elevate SAM levels and induce *var2csa* expression, whereas methionine depletion produces *var* expression profiles akin to those observed under standard culture conditions. Additional evidence for the central role of SAM in the regulation of *var* gene expression comes from experiments in which the expression of SAM synthetase (PfSAMS) was enhanced. As PfSAMS catalyzes the conversion of methionine and ATP into SAM, its overexpression mirrors the effects of exogenous methionine or choline supplementation by elevating intracellular SAM levels and promoting the activation of *var2csa* [42]. Collectively, these findings support a comprehensive model in which the metabolic state of *P. falciparum*, particularly the SAM/SAH ratio, is a decisive factor in the regulation of *var* gene expression [42,43]. Environmental fluctuations that affect the availability of lipid precursors or methionine lead to alterations in SAM production and consumption; elevated SAM levels enhance the activity of histone methyltransferases that deposit activating marks, thereby perturbing the epigenetic equilibrium at *var* loci and resulting in the derepression of *var2csa* and potentially other *var* genes [36,40,42]. Conversely, conditions that maintain or diminish SAM levels serve to reinforce the repressive chromatin state, preserving the mutually exclusive expression pattern vital for antigenic variation.

### 3.3. Implications for Host–Parasite Interactions and Therapeutic Approaches

The notion that *var2csa* may represent a ‘default’ switch point within the *var* gene network is particularly compelling. Its distinctive promoter architecture and translational regulation mediated by an upstream open-reading frame suggest that *var2csa* occupies a central role in the regulatory circuitry governing *var* gene switching [40,41]. The consistent activation of *var2csa* under conditions of elevated SAM implies that this gene may function as a sensor of metabolic stress, enabling the parasite to swiftly adjust its antigenic profile in response to environmental changes within the host [38,39]. The interdependence of metabolic cues and epigenetic regulation in *P. falciparum* has profound implications for our understanding of host–parasite interactions. The ability of the parasite to modulate *var* gene expression in response to nutrient availability exemplifies its remarkable adaptability, allowing it to balance the competing demands of immune evasion and rapid replication with the substantial metabolic requirements of membrane biosynthesis [52,53,54,55,56,57]. Moreover, the intricate linkage between lipid metabolism and epigenetic control emphasizes the extent to which the host environment can influence parasite virulence, as fluctuations in the host’s metabolic status, whether due to fever or nutritional stress, can directly affect the parasite’s epigenetic landscape and antigenic expression [53,54,61]. This sophisticated interplay between metabolism and epigenetics also presents novel opportunities for therapeutic intervention. Inhibiting key enzymes within the one-carbon metabolic pathway, such as PfPMT or PfSAMS, might disrupt the finely tuned regulation of *var* gene expression and, by extension, compromise the parasite’s capacity for immune evasion [42,60,62,63]. Such strategies are especially attractive in the context of rising drug resistance. The body of evidence thus underscores the central importance of the intracellular SAM/SAH ratio in orchestrating *var* gene regulation in *P. falciparum* [42,43,44]. The mutually exclusive expression of *var* genes, underpinned by a complex network of histone modifications, is intricately modulated by the parasite’s one-carbon metabolism. Environmental variations, particularly in choline, serine and methionine levels, directly impact the SAM/SAH ratio and, consequently, the activity of histone methyltransferases, thereby precipitating a coordinated switching of *var* gene expression with *var2csa* emerging as a key regulatory element [42,43]. This integration of metabolic signals into the epigenetic machinery equips *P. falciparum* with a rapid and adaptive mechanism to modulate its antigenic profile in response to host environmental fluctuations, ensuring its survival and virulence [36,40,52,53,54,55,56,57]. These insights not only deepen our understanding of malaria pathogenesis but also highlight promising metabolic targets for future therapeutic interventions.

## 4. Co-Adaptive Dynamics with Human and Mosquito Hosts

Malaria remains a significant global health concern, driven by the complex co-evolutionary relationship between the *Plasmodium falciparum* parasite, its human hosts, and its mosquito vectors. This intricate interplay has spurred the development of sophisticated genetic and immune defenses in human populations. Simultaneously, the parasite’s success is not only dependent on navigating the human host but also on its specific adaptation to its invertebrate vectors. Gaining a deeper insight into these adaptations is vital for crafting effective, long-term strategies to fight malaria. Understanding the parasite’s reliance on the mosquito, for instance, has paved the way for promising preventive strategies like transmission blocking vaccines. A comprehensive approach that considers the full triad of human, parasite, and vector interactions is therefore essential for developing further solutions.

### 4.1. Human Host Adaptations and Immune Dynamics

The intractable and globally consequential challenge of malaria stems from a source far deeper than the inherent biological complexity of *P. falciparum* parasites; it is profoundly and irrevocably enmeshed within the intricate, millennia old co adaptive dance between parasite, human host, and mosquito vector. This tritrophic evolutionary drama, played out across vast ecological landscapes and spanning countless generations, has sculpted a system of astonishing plasticity and evolutionary resilience, rendering malaria a persistent and formidable antagonist in the global struggle for health equity. A truly nuanced and predictive understanding of these complex adaptive dynamics, characterized by a relentless and reciprocal exchange of selective pressures amongst each participant, is not merely academically valuable, but constitutes an existential imperative for the design and implementation of intervention strategies that transcend the limitations of transient disease management and aspire to the transformative goal of sustainable eradication. The human host, serving as both the definitive incubator for parasite reproduction and the ultimate stage for its pathological drama, has, through the unforgiving crucible of natural selection, evolved a remarkably sophisticated and multifaceted repertoire of defenses against malarial invasion. While the foundational insights of pioneering researchers like Haldane and Livingstone, though predating the modern genomic era, remain conceptually relevant, contemporary investigations have unveiled a far more intricate and spatially heterogeneous landscape of human adaptation. Beyond the hemoglobinopathies, such as sickle cell trait and thalassemia, which stand as enduring testaments to the power of balancing selection [64,65], a burgeoning body of evidence now points to a diverse spectrum of genetic polymorphisms, encompassing erythrocyte structural variants, immunomodulatory gene loci, and metabolic adaptations, that collectively contribute to a complex and geographically mosaic pattern of human resistance to malaria [65]. Furthermore, the acquisition of functional immunity in human populations repeatedly exposed to *P. falciparum* infection remains a cornerstone of malarial epidemiology, a testament to the remarkable plasticity of the human immune system. This acquired immunity, while demonstrably imperfect in its capacity to confer sterile protection and characterized by a frustrating tendency towards temporal decay [66], is nevertheless of profound clinical significance, demonstrably mitigating disease severity, substantially attenuating morbidity, and dramatically reducing malaria attributable mortality, particularly in adult cohorts residing within endemic regions. This complex immunological response represents a finely orchestrated symphony of humoral and cellular effector mechanisms, meticulously targeting a bewildering array of parasite antigens expressed across its ontogenetically diverse life cycle [67,68]. However, the human immune system, far from representing a static and impenetrable fortress, exists in a perpetual state of dynamic evolutionary tension, relentlessly evolving and perpetually being molded by the adaptive ingenuity of the parasite itself. The parasite’s truly extraordinary and arguably unparalleled capacity for antigenic variation, most spectacularly exemplified by the seemingly limitless repertoire of *var* genes encoding the immunodominant PfEMP1 protein in *P. falciparum* [69,70], constitutes a masterful evolutionary innovation, effectively enabling parasites to strategically evade pre-existing, memory-based antibody responses, and perpetually necessitating the energetically costly and immunologically demanding de novo generation of novel adaptive immune responses within the perpetually challenged human host population. This breathtaking and seemingly inexhaustible antigenic diversity, when intricately coupled with the parasite’s equally sophisticated and clinically relevant arsenal of active immunomodulatory mechanisms, allowing it to strategically suppress, actively subvert, and subtly manipulate protective host immune responses [71], synergistically contributes to the chronic, often relapsing, and therapeutically recalcitrant nature of malarial infection, and continues to confound and frustrate the long standing and intensely pursued quest for a truly sterilizing and universally effective malaria vaccine.

### 4.2. Mosquito Vector Adaptations and Insecticide Resistance

In parallel with the dynamic human–parasite interaction, the mosquito vector, represented primarily by species within the medically and epidemiologically significant *Anopheles* genus, serving as the indispensable definitive host in the obligate *P. falciparum* life cycle, has also been subjected to similarly intense and transformative co-evolutionary pressures, albeit driven by distinct environmental and anthropogenic forces. Mosquito physiology and, critically, mosquito behavioral ecology exert a direct and quantifiable influence on vectorial capacity—the ultimate epidemiological determinant of a vector population’s efficiency in mediating malaria transmission within a defined ecological and epidemiological context [72]. Vector competence, rigorously defined as the intrinsic physiological capacity of a specific mosquito species or strain to permissively support the complete developmental cycle of a *P. falciparum* parasite, encompassing sporogony and culminating in the efficient transmission of infectious sporozoites, is a complex and polygenic phenotype, governed by a dynamic and context dependent interplay of host genetic predisposition and environmental variables [73,74]. The genetic architecture of the mosquito vector is demonstrably paramount in modulating its intrinsic susceptibility to *P. falciparum* infection, with specific genes demonstrably exerting fine grained control over parasite invasion of the midgut epithelial barrier, developmental progression through the intricate sporogonic cycle within the mosquito hemocoel and salivary glands, and ultimately, the quantitative efficiency of sporozoite dissemination and subsequent transmission to a susceptible vertebrate host during hematophagy [75,76]. However, arguably the most globally significant and clinically consequential facet of mosquito adaptation in the contemporary era has been the alarmingly rapid, geographically expansive, and now demonstrably entrenched evolution of insecticide resistance. The sustained, widespread, and often indiscriminate application of synthetic insecticides, particularly the cost-effective and historically efficacious pyrethroid class, as the cornerstone of global malaria vector control interventions, has inadvertently imposed a directional and profoundly potent selective pressure upon global mosquito populations, inexorably driving the relentless and accelerating emergence and subsequent global dissemination of a truly bewildering diversity of insecticide resistance mechanisms, both physiological and behavioral [77,78]. These resistance mechanisms, reflecting the remarkable adaptability of insect genomes and the multifaceted nature of evolutionary adaptation to anthropogenic pressures, are exceptionally diverse, encompassing metabolic detoxification mechanisms, wherein mosquito populations evolve enhanced and often constitutively upregulated enzymatic machinery to efficiently catabolize and biochemically neutralize insecticide molecules before they reach their neuronal targets [79], and target site insensitivity mutations, which subtly but significantly alter the molecular architecture of the insecticide’s cognate binding domain within the mosquito nervous system, thereby diminishing its pharmacological affinity, reducing its bioactivity, and ultimately compromising its entomological efficacy [80]. The clinically dire consequence of this escalating global tide of insecticide resistance is a demonstrably quantifiable and epidemiologically significant erosion in the protective efficacy of insecticide-treated bed nets (ITNs) and indoor residual spraying (IRS), previously considered foundational and remarkably effective interventions in global malaria control, now posing a tangible and accelerating threat to undermine decades of hard won public health gains and potentially reversing previously hard fought progress towards the ambitious global malaria elimination agenda [81]. Transcending insecticide resistance sensu stricto, mosquito populations also exhibit a remarkable and often underappreciated capacity for sophisticated behavioral adaptation, enabling them to strategically circumvent conventional control pressures and enhance their overall survival and vectorial capacity within increasingly human modified and intervention saturated landscapes. These clinically relevant behavioral adaptations encompass striking temporal plasticity in host-seeking behavior, including demonstrably pronounced and geographically structured shifts in biting chronologies, transitioning from historically dominant endophilic (indoor) and nocturnal biting patterns to increasingly prevalent exophilic (outdoor) and crepuscular or even diurnal biting behaviors, thereby strategically reducing vector-human contact and effectively minimizing exposure to indoor based vector control interventions such as ITNs and IRS [82,83]. Furthermore, mosquito populations are increasingly demonstrating an adaptive capacity to develop active avoidance behaviors, exhibiting learned aversion to insecticide-treated surfaces, and even physically avoiding contact with bed nets, further eroding the protective efficacy of these historically pivotal vector control tools and necessitating the development of innovative and behaviorally informed intervention strategies [84]. Recent advances in malaria control have identified transmission-blocking vaccines (TBVs) as a promising complementary intervention targeting the parasite’s development within the mosquito vector. TBVs function by inducing host immune responses that produce antibodies against sexual-stage Plasmodium antigens [85]. Upon ingestion by mosquitoes during blood feeding, these antibodies disrupt parasite fertilization and subsequent development in the mosquito midgut, thereby reducing the formation of infectious sporozoites. This biological blockade effectively decreases mosquito infectivity and vectorial capacity without relying on insecticidal activity or mosquito behavior modification [85]. Consequently, TBVs present a strategic innovation capable of circumventing the growing challenges posed by insecticide resistance and behavioral avoidance in mosquito populations. Integration of TBVs into existing vector control frameworks may significantly enhance malaria transmission interruption efforts and accelerate progress toward malaria elimination goals.

## 5. Conclusions

In summary, Plasmodium falciparum evolutionary history is a careful series of adaptations that, taken together, shaped these organisms into obligatory intracellular parasites. These adaptations ranged from the divergence of a photosynthetically competent progenitor to the acquisition and repurposing of the apicoplast, to significant genome reduction, and to frequent horizontal gene transfers. This trajectory culminated in a highly sophisticated pathogenic strategy where the parasite’s ability to seamlessly integrate its one-carbon metabolism with its chromatin architecture is essential to its success. PfEMP1 variants are expressed in a mutually exclusive manner, and subtle changes in the intracellular SAM/SAH ratio, which are controlled by the coordinated activity of SET-domain methyltransferases (PfSET1, PfSET2, PfSET3, and PfSET10), result in precise histone methylation at various loci. With its dual transcriptional–translational control, *var2csa* stands out among these as a crucial metabolic-sensing node that can quickly switch antigens in response to changes in nutrient availability. This epigenetic framework works in tandem with host polymorphisms, such as immunomodulatory alleles and hemoglobinopathies, as well as Anopheles vector adaptations, such as behavioral changes and insecticide resistance, and the unrelenting accumulation of antimalarial drug-resistant mutations. To address the persistent problem of malaria, future research must adopt an integrated approach. This includes not only exploiting apicoplast-dependent biosynthetic pathways and one-carbon enzymes as chemotherapeutic targets but also dissecting the interface between metabolic flux and chromatin remodeling in molecular detail. Emerging evidence highlights an interplay of methyltransferases and putative demethylases in *P. falciparum*, suggesting a potentially reversible epigenetic regulation—though specific counteracting demethylases remain to be characterized [27]. Understanding such plasticity at the molecular level complements systems-level surveillance, supports the development of next-generation vaccines that target multiple life stages, and informs vector control strategies that account for behavioral adaptation. Only by integrating these mechanistic insights into interdisciplinary approaches can we achieve the precise interventions required to eradicate malaria.

## Figures and Tables

**Table 1:** Functional Roles of the Apicoplast in Plasmodium falciparum. This table summarizes the major metabolic functions retained by the apicoplast in Plasmodium falciparum, highlighting its essential role in parasite survival and its potential as a drug target.

Metabolic Pathway	Products/Functions	Therapeutic Relevance
Fatty acid biosynthesis	Precursors for membrane lipids	Inhibitors block parasite growth in liver stage
Isoprenoid biosynthesis	Essential for protein prenylation	Fosmidomycin targets this pathway
Haem biosynthesis	Cofactor for electron transport	Loss impairs survival under stress conditions
tRNA and protein synthesis	Supports apicoplast-encoded functions	Disruption → delayed death phenotype

**Table 2:** Epigenetic Regulators of *var* Gene Expression. Key histone modifications and regulatory proteins that control var gene expression in Plasmodium falciparum, linking metabolism to antigenic variation.

Epigenetic Mark/Regulator	Role in *var* Gene Regulation	Connection to Metabolism
H3K9me3 (trimethylation)	Maintains silent heterochromatin at subtelomeric var loci	Requires SAM as methyl donor
H3K9ac (acetylation)	Marks active var promoter regions	Balance shifts with nutrient availability
HP1 (Heterochromatin Protein 1)	Mediates clustering of silent var genes at nuclear periphery	Sensitive to chromatin methylation state
PfSET10 (histone methyltransferase)	Maintains active var gene in poised state	Activity depends on SAM pool
